# Osteopenia in a Mouse Model of Spinal Cord Injury: Effects of Age, Sex and Motor Function

**DOI:** 10.3390/biology11020189

**Published:** 2022-01-26

**Authors:** Michelle A. Hook, Alyssa Falck, Ravali Dundumulla, Mabel Terminel, Rachel Cunningham, Arthur Sefiani, Kayla Callaway, Dana Gaddy, Cédric G. Geoffroy

**Affiliations:** 1 Department of Neuroscience and Experimental Therapeutics, College of Medicine, Texas A&M Health Science Center, Bryan, TX 77807, USA; ravalidundumulla@gmail.com (R.D.); mabelt19@gmail.com (M.T.); rcunnink@gmail.com (R.C.); sefiani@tamu.edu (A.S.); kjcallaway33@tamu.edu (K.C.); geoffroy@tamu.edu (C.G.G.); 2 Veterinary Integrative Biosciences, College of Veterinary Medicine and Biomedical Sciences, College Station, TX 77843, USA; afalck@cvm.tamu.edu (A.F.); dgaddy@cvm.tamu.edu (D.G.)

**Keywords:** spinal cord injury, osteopenia, bone loss, recovery of function

## Abstract

**Simple Summary:**

In the first two years following spinal cord injury, people lose up to 50% of bone below the injury. This injury-induced bone loss significantly affects rehabilitation and leaves people vulnerable to fractures and post-fracture complications, including lung and urinary tract infections, blood clots in the veins, and depression. Unfortunately, little is known about the factors driving this bone loss. In fact, even though we know that injury, age, and sex independently increase bone loss, there have been no studies looking at the cumulative effects of these variables. People with spinal injury are aging, and the age at which injuries occur is increasing. It is essential to know whether these factors together will further compromise bone. To examine this, we assessed bone loss in young and old, male and female mice after spinal injury. As expected, we found that aging alone decreased motor activity and bone volume. Spinal injury also reduced bone volume, but it did not worsen the effects of age. Instead, injury effects appeared related to reduced rearing activity. The data suggest that although partial weight-bearing does not reduce bone loss after spinal cord injury, therapies that put full weight on the legs may be clinically effective.

**Abstract:**

After spinal cord injury (SCI), 80% of individuals are diagnosed with osteopenia or osteoporosis. The dramatic loss of bone after SCI increases the potential for fractures 100-fold, with post-fracture complications occurring in 54% of cases. With the age of new SCI injuries increasing, we hypothesized that a SCI-induced reduction in weight bearing could further exacerbate age-induced bone loss. To test this, young (2–3 months) and old (20–30 months) male and female mice were given a moderate spinal contusion injury (T9–T10), and recovery was assessed for 28 days (BMS, rearing counts, distance traveled). Tibial trabecular bone volume was measured after 28 days with ex vivo microCT. While BMS scores did not differ across groups, older subjects travelled less in the open field and there was a decrease in rearing with age and SCI. As expected, aging decreased trabecular bone volume and cortical thickness in both old male and female mice. SCI alone also reduced trabecular bone volume in young mice, but did not have an additional effect beyond the age-dependent decrease in trabecular and cortical bone volume seen in both sexes. Interestingly, both rearing and total activity correlated with decreased bone volume. These data underscore the importance of load and use on bone mass. While partial weight-bearing does not stabilize/reverse bone loss in humans, our data suggest that therapies that simulate complete loading may be effective after SCI.

## 1. Introduction

In the first two years following a spinal cord injury (SCI), there is a 30–50% decrease in bone mineral density in the lower limbs [1,2,3,4]. This demineralization occurs primarily in the trabecular bone found in the distal and proximal epiphysis and metaphysis of the femur and tibia, respectively [5,6,7]. Albeit more slowly, decreases in cortical bone mineralization and thinning of the cortical bone walls also occur [3,8,9,10], rendering the lower limbs susceptible to fracture. More than 30% of adults living with SCI sustain fragility fractures of the lower extremities [11,12,13,14,15,16], an incidence two-fold greater than in the able-bodied population, with most fractures occurring during normal daily living activities (i.e., transferring to and from a wheelchair, dressing, and bathing [17]). These fractures not only compromise rehabilitation, they also increase morbidity, mortality, and healthcare costs. Alarmingly, post fracture complications also occur in 50% of SCI patients [15,18,19]. These complications include, but are not limited to, respiratory and urinary tract infections, venous thromboembolic events, fracture non-union or mal-union, and depression. Bone loss after SCI significantly reduces a person’s quality of life and compromises physical well-being.

Numerous factors have been implicated in SCI-induced bone loss. First, there is loss of signaling from muscle to bone. There is rapid loss of muscle after SCI: with muscle weights reduced by 40–60% in young male rats just two weeks after a spinal cord transection [20]. Normally, during muscle contractions and weight bearing, muscles release signaling molecules including myokines that modulate the activity of osteoblasts and osteoclasts, thereby affecting bone formation and resorption respectively [21,22,23]. For example, the recently discovered myokine irisin is reported to increase osteoblast differentiation and suppress osteoclast activity [24,25,26], although evidence suggests that its effects may be dose and duration of exposure dependent [27]. Second, the disruption of sympathetic neuronal signaling with SCI is posited to affect bone health by, e.g., allowing intravenous shunts to open throughout bone, reducing blood flow through the tissue. The resulting vascular stasis leads to decreased gas and nutrient exchange, and promotion of local hyper-pressure which triggers osteoclast formation [28]. Dysfunction of the pituitary-hypothalamic axis [29,30,31], increased adiposity in the bone marrow and muscle [22,32,33,34,35], as well as insulin resistance [22], inflammation [36], and deficiencies of Vitamin D [37] also contribute to bone loss after SCI. The multifactorial effects of SCI ultimately result in bone formation being uncoupled from bone resorption, with a reduction in bone mass and decreased skeletal integrity.

Along with SCI, aging and time post-injury exponentially increase fracture risk [38,39]. In the able-bodied population, bone mass peaks at about 25–30 years and then begins to decline [40,41]. In fact, by 50–59 years of age, it is estimated that 5.1% of individuals in this age group have osteoporosis, and an additional 40.2% have low bone mass [42]. Pre-existing low bone mass and osteoporosis subsequently render older individuals vulnerable to SCI, with an increasing incidence of injuries due to falls occurring in people older than 60 years [43,44,45,46,47,48]. Superimposing SCI onto an already compromised system further compounds the risk for fracture and related complications.

Similarly, changes in levels of sex hormones with age affect bone loss. After 50 years of age, the incidence of osteoporosis and low bone mass is more than three times greater in females than in males [49,50], an effect attributed to the rapid loss of estrogen during menopause [51,52,53]. The effects of estrogen on bone are particularly relevant for SCI. Amenorrhea and perimenopause symptoms are frequently observed in the acute phase of SCI in both humans and animal models [54,55,56]. SCI coupled with an acute decrease in estrogen levels, and prolonged decreases with aging, would significantly compromise bone health. While the majority of traumatic SCIs occur in males, this demographic is changing particularly in older age groups [45,46,47,57]. It is essential that we develop strategies to reduce the loss of bone seen in the early stages of SCI and facilitate the rebuilding of bone in chronic stages for all age and sex groups.

Surprisingly, however, despite significant effects of SCI, age, and sex on bone loss, there have been no studies looking at the interactive effects on bone in an SCI model. To address this, the current study assessed bone loss in young and old, male and female mice after a moderate spinal contusion injury. We recorded locomotor function, activity levels in an open field, and rearing for one month post injury. At the end of recovery, cancellous and compact bone was compared across groups. Commensurate with clinical findings, we found significant effects of age and sex on bone loss, as well as age-dependent positive correlations between bone loss and rearing activity.

## 2. Methods

### 2.1. Subjects

Both young (2–3 months) and old (20–30 months) male and female C57BL/6 mice served as subjects. In total, 15 young males (4 shams, 11 SCI), 15 young females (7 shams, 8 SCI), 9 old males (4 shams, 5 SCI), and 16 old females (8 sham, 8 SCI) were available in our Texas A&M Health Science Center animal facility for transfer to this animal use protocol and assessment in this study. The mice were housed with littermates in ventilated cages and maintained on a 12 h light-dark cycle, with access to both food and water ad libitum. Mixed groups of aged and sex-matched SCI and sham mice were housed together, with their positions on the cage racks randomized across groups. All testing and surgeries occurred during the light cycle. Following a contusion injury, subjects’ bladders were expressed manually every morning between 7:00 and 9:00 a.m. and evening between 4:30 and 6:30 p.m. This schedule was maintained until subjects regained bladder function (operationally defined as voiding on own for three consecutive days). All of the experiments reported here were reviewed and approved by the Institutional Animal Care Committee at Texas A&M and were consistent with the NIH guidelines for animal care and use.

### 2.2. Spinal Contusion Injury

The mice were given a moderate spinal contusion injury (50 kdyne, T9–T10, Infinite Horizons Impactor), or served as sham-injured controls. Briefly, under deep anesthesia with isoflurane (2% gas, Piramal Pharma Solutions, Telangana, India), a small skin incision was made over the spinal cord column covering lamina T7–T12. The spinous process of T9 was located, and the T9–T10 vertebrae were removed (laminectomy). For the spinal contusion injury, the vertebral column was fixed within the IH device, using two Adson forceps, and a controlled impact was delivered to the exposed cord with a stainless steel impactor tip. The mouse was visually inspected for evidence of bruising of the cord, and then the muscles were sutured and the skin closed with Dermabond. All mice received daily injections of saline (0.9%) and buprenorphine (0.05 mg/kg, Par Pharmaceutical Chestnut Ridge, Chestnut Ridge, NY, USA) for 3 days and penicillin (5 mg/kg/day, Bayer Healthcare LLC, Animal Health Division Shawnee Mission, Shawnee, KS, USA) for 7 days. Mice were monitored daily, with their bladder expressed manually twice every day until the mice were able to urinate without assistance or till the end of the experiment. As the focus of this experiment was on spinal cord injury effects, rather than other stressors associated with handling, anesthesia or peripheral injury that could affect bone health [58,59], sham-injured mice served as controls. Sham mice underwent all of the same surgical and general husbandry procedures as the spinal contusion mice with the exception of the controlled impact to the exposed spinal cord, enabling the assessment of the contusion effects on bone and activity independent of general surgery/recovery.

### 2.3. Assessment of Motor Recovery

Locomotor function was assessed for 28 days post injury using the Basso Mouse Scale (BMS [60]). This test measures hindlimb motor movement with scores ranging from slight ankle movement (BMS = 1) to occasional plantar weight supported stepping (BMS = 4) and coordinated walking (BMS = 9). BMS scores were collected on the day following injury, and then every other day from days 1–13 post injury, and every 3rd day from day 16 until day 28 post-injury. Mice were placed in the open enclosure (120 cm long, 60 cm wide, 23 cm deep) and observed for 4 min. Experimenters were kept blind to subject’s experimental treatment. Subjects were excluded from the experiment if BMS scores were >3 on the day following injury.

Motor recovery was also assessed with a rearing test. The mice were placed in a high-ceiling Plexiglas chamber (27 cm long, 15 cm wide, 17 cm high) for 5 min, in a quiet dark room (with red light), and videotaped. The number of rears performed in the 5-min session was determined from post hoc video analyses. Rearing activity was assessed prior to surgery, and then every 7th day until day 28 post-injury.

Open field activity levels were also assayed in automated activity chambers (43 cm long, 43 cm wide, 15 cm deep, Hamilton-Kinder LLC, Chula Vista, CA, USA). Activity was detected as infrared beam crosses (2.5 cm spacing). Each mouse was placed in the center of an open field chamber and allowed to explore the apparatus freely for 10 min. The distance traveled within the study time-period was recorded. Open field activity was assessed prior to surgery and every 7 days until day 28.

At the end of the recovery period, the mice were deeply anesthetized (100 mg/kg of beuthanasia, intraparietal) and perfused intracardially with PBS and 4% paraformaldehyde. Following perfusion, a 1 cm segment centered at the lesion site was extracted and prepared for cryostat sectioning. Sagittal sections (25 µm) were collected and stained as follows. Sections were washed (3× with 0.4% Triton X-100 in 1× PBS), then blocked using 5% normal horse serum (VWR 102643-676, Radnor, PA, USA), diluted in 0.4% Triton X-100 in 1× PBS, for 1 h. Sections were then incubated with primary anti-glial fibrillary acidic protein (GFAP) antibody (1:500, Cat. No. 13-0300, ThermoFisher Scientific, Waltham, MA, USA), diluted in 1× PBS with 0.4% Triton X-100 (PBS-TX), at 25 °C overnight. The sections were then washed 3 times in PBS-TX before being incubated with Alexa Fluor Plus 488 secondary antibody (1:1000, Cat. No. A32814, ThermoFisher Scientific) in PBS-TX for 1 h and DAPI (1 μg/mL, Cat. No. 62248, ThermoFisher Scientific) for 5 min. After being washed once more (PBS), the slices were mounted onto Superfrost Plus slides and coverslipped (Cat. No. F6182, Sigma, St. Loius, MO, USA). Sections were imaged at 10× on a Zeiss Axio Observer 7 fluorescent microscope. The contour (polygonal) tool in Zen 3.2 (Carl Zeiss AG, Oberkochen, Germany) software was used to trace and measure lesion size, tracing the glial scar border labeled with GFAP surrounding a DAPI+ inner region. Three spinal cord sections per mouse were imaged and analyzed.

### 2.4. Tibial MicroCT Analysis

Tibiae were harvested at sacrifice and fixed in neutral buffered formalin prior to evaluation of trabecular bone volume and architecture as well as cortical bone geometry by micro-computed tomography (µCT50, Scanco Medical, Brüttisellen, Switzerland). Briefly, the proximal tibia and tibial midshaft regions were scanned as 12 µm isotropic voxel size using 55 kVp, 114 mA, and 200-ms. Bone volume fraction (BV/TV, %), trabecular thickness (Tb.Th, mm), trabecular separation (Tb.Sp, mm), trabecular number (Tb.N, 1/mm), connectivity density (ConnD 1/mm^3^), and volumetric bone mineral density (BMD, mg/mm^3^) were calculated using previously published methods [61]. The cancellous bone region was obtained using a semi-automated contouring program that separated cancellous from cortical bone. At the midshaft, of the tibia, total cross sectional area (CSA, mm^2^), cortical thickness (Ct.Th, mm), periosteal perimeter (mm), and endosteal perimeter (mm) were assessed in a 1 mm long region. Bone was segmented from soft tissue using the same threshold for all groups, 245 mg HA/cm^3^ for trabecular and 682 mg HA/cm^3^ for cortical bone. All microCT scanning and analyses were compliant with published American Society for Bone and Mineral Research (ASBMR) guidelines for rodents [62]. For the assessment of bone, the investigators were kept blind to all subject’s experimental treatment. Age, sex, and injury condition were revealed at the end of all assessments.

### 2.5. Statistical Analyses

Three-way repeated measure ANCOVAs were used to compare locomotor recovery, open field activity and rearing across groups. In these analyses, Day 1 BMS scores served as covariates in tests of locomotor function, and pre-injury assessments of rearing and open field activity were covariates in these respective analyses. In all tests, day post injury served as the repeated measure. Significant main effects (*p* < 0.05) of age, sex, or injury were further analyzed with independent t-tests to examine temporal effects on behavior.

Three-way ANOVAs were also used to compare trabecular bone volume and architecture, and cortical bone geometry across groups, followed by post hoc independent t-tests comparing the means of groups that differed by only one factor (age, sex, or injury). Differences were considered significant when *p* < 0.05. All data analyses were performed using SPSS version 27.0 (SPSS, Inc., Chicago IL, USA).

## 3. Results

### 3.1. The Effects of SCI on Motor Recovery Are Task Dependent

**Figure 1:**
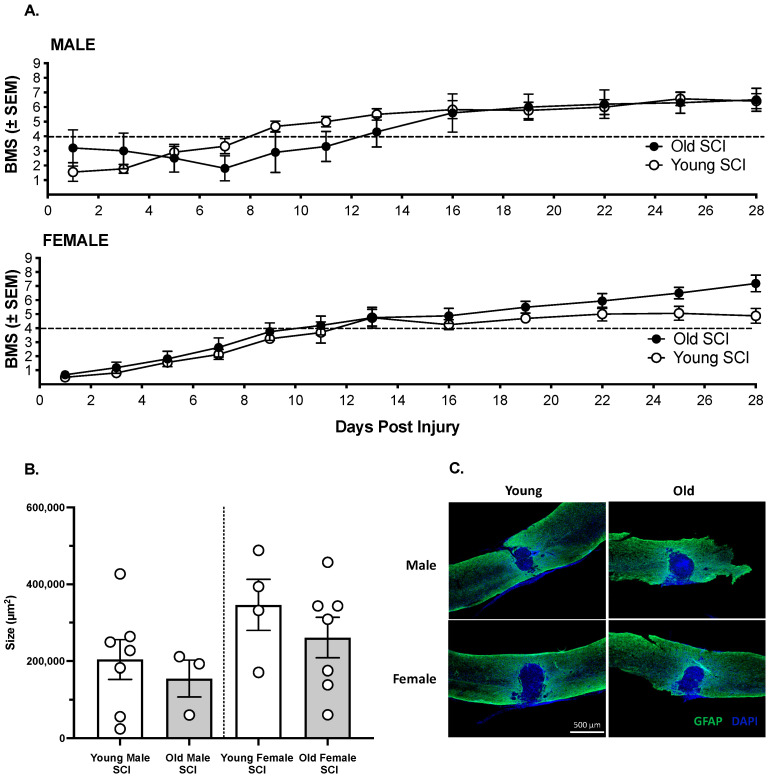
(**A**). Irrespective of age or sex all mice recovered locomotor function, assessed with the BMS scale, over 28 days post injury. There were no significant differences in recovery across groups. The Mean ± SEM for each group is shown across days, and the dashed line represents the score for plantar, weight supported stepping. (**B**). Similarly, age and sex did not affect lesion size at 28 days post injury. The white circles represent individual subjects. (**C**). Representative images of the lesion, visualized with GFAP and DAPI staining, from each demographic are shown. For BBB analyses (**A**), n = 5 old males, 11 young males, 8 old females, 8 young females. For lesion analyses (**B**), n = 3 old males, 6 young males, 7 old females, 4 young females.

All sham-operated subjects had the maximum BMS score of 9 across days. SCI significantly decreased locomotor function. Further examining the SCI subjects only, a three-way ANCOVA revealed no effect of sex (F (1,27) < 0.01, *p* > 0.05) or age (F (1,27) < 0.01, *p* > 0.05) on the recovery of locomotor function (Figure 1A). All groups recovered plantar weight supported stepping by Day 14 post injury. Analyses of the lesion size at 28 days post injury also revealed no significant differences between groups (F (3,17) = 1.51, *p* > 0.05, Figure 1B,C).

**Figure 2:**
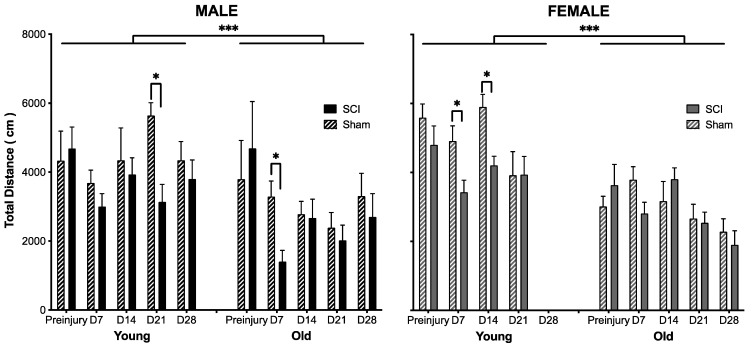
Age, sex and SCI affected open field activity. The young mice displayed greater activity in the open field than the old mice, with females also displaying increased activity relative to males. Not surprisingly, there was a main effect of SCI on activity. SCI reduced activity relative to sham controls, particularly in the early stage of injury (Day 7), irrespective of the demographic observed. Days (D) post injury are denoted on the x-axis, with the mean (±SEM) distance traveled in 10 min depicted on the y-axis. n = 4 sham and 5 SCI old males; 4 sham and 11 SCI young males; 8 sham and 7 SCI old females; 7 sham and 8 SCI young females. *** *p* < 0.0001 and * *p* < 0.01.

Open field activity, however, was affected by injury, sex, and age (Figure 2). Not surprisingly, subjects given a sham injury displayed increased open field activity relative to SCI subjects (F (1,46) = 12.48, *p* < 0.001). This effect was largely driven by reduced activity in all groups during the early phase of SCI. There was an effect of surgery on activity on Day 7 post injury (*t* = −3.64, *p* < 0.001), but no differences were observed across groups on Days 14–21 (*t* < −1.09, *p* > 0.05, on all days). Statistical comparisons were not performed for Day 28, because the data for young females were corrupted and could not be included. As can be seen in Figure 2, however, there were no differences in open field activity for sham and contused mice in the remaining groups on Day 28. Similarly, the females displayed increased open field activity relative to males on Day 7 post injury (*t* = 2.45, *p* < 0.02), but at no other timepoint. Irrespective of the timepoint post injury, young mice displayed greater open field activity than old mice (*t* > 2.04, *p* < 0.05 for all comparisons).

**Figure 3:**
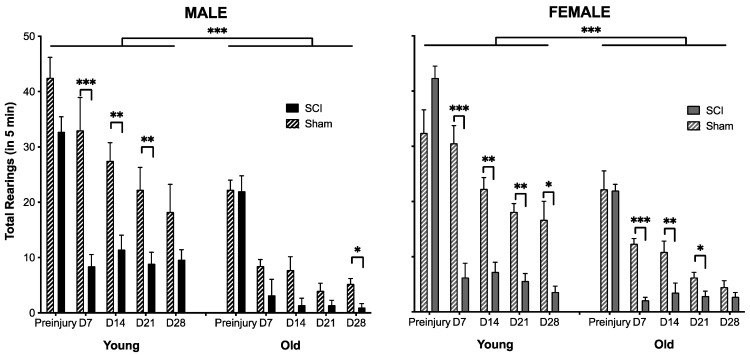
Age and SCI affected rearing activity. The young mice reared more than the old mice and SCI reduced rearing activity relative to sham controls, irrespective of the demographic observed. There was no effect of sex on rearing behavior. Days (D) post injury are denoted on the x-axis, with the mean (±SEM) number of rears in 5 min shown on the y axis. n = 4 sham and 5 SCI old males; 4 sham and 11 SCI young males; 8 sham and 7 SCI old females; 7 sham and 8 SCI young females. *** *p* < 0.0001, ** *p* < 0.001, * *p* < 0.01.

As shown in Figure 3, there was also a significant main effect of injury (F (1,46) = 63.99, *p* < 0.001) and age (F (1,46) = 21.33, *p* < 0.001), but no effect of sex (F (1,46) < 1.0, *p* > 0.05) on rearing. There was also a significant interaction between injury and age (F (1,46) = 16.95, *p* < 0.001). Rearing was decreased in SCI subjects at all timepoints post injury, relative to sham controls (*t* > 2.58, *p* < 0.01 in all comparisons). Similarly, young subjects reared more than old subjects at all days post injury (*t* > 3.14, *p* < 0.005, for all comparisons). It is also noted that the sham subjects decreased rearing across testing days, likely reflecting decreased exploration as the experimental testing chamber became more familiar across sessions.

### 3.2. SCI Significantly Increases Bone Loss

**Figure 4:**
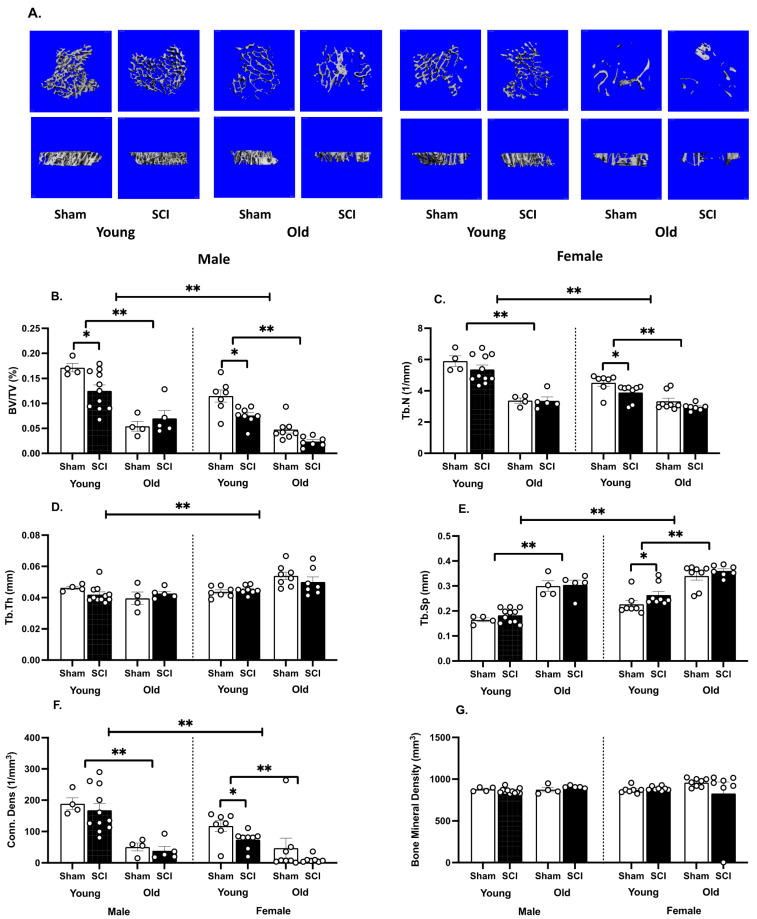
SCI, age, and sex affected trabecular bone. (**A**). Coronal (**top** panels) and lateral (**bottom** panels) view of microCT 3D reconstructions of 200 contiguous slices of the tibial metaphysis from a representative mouse in each group. (**B**). Females has less trabecular volume than males and, irrespective of sex, trabecular volume decreased with age. For the young subjects, SCI also decreased trabecular volume. (**C**). Females also had a reduced number of trabeculae, and the number of spicules was lower in older subjects regardless of sex. In young females there was also a decrease in trabecular number with age. (**D**). There was a main effect of sex on trabecular thickness, with females showing increased thickness relative to males. Age and sex did not affect this parameter. (**E**). Sex, age and SCI affected trabecular spacing. Spacing increased in females, relative to males, and in older subjects relative to young. SCI increased trabecular spacing in the young females. (**F**). Connective density was also greater in males relative to females, and in young compared to old mice. SCI decreased connective density in young females. (**G**). There was no effect of sex, age or SCI on bone mineral density. Means ± SEMs are plotted for each group. Individual subjects are shown with the round, white symbols. n = 4 sham and 5 SCI old males; 4 sham and 11 SCI young males; 8 sham and 7 SCI old females; 7 sham and 8 SCI young females. ** *p* < 0.01, * *p* < 0.05.

A three-way ANOVA revealed main effects of age (F (1,46) = 88.7, *p* < 0.0001), sex (F (1,46) = 26.36, *p* < 0.0001) and injury (F (1,46) = 9.12, *p* < 0.005) on trabecular bone volume (Figure 4A,B), as well as a significant interaction between injury and age (F (1,46) = 6.14, *p* < 0.05). Irrespective of sex and injury, old mice had lower trabecular bone volume than young mice (*t* > 3.75, *p* < 0.0005 for all comparisons). The young females also had lower trabecular bone remaining compared with the young males (*t* = 3.36, 3.95, for Sham and SCI subjects respectively, *p*< 0.002). There was no difference between trabecular bone volume for old sham males compared with old sham females (*t* = 0.40, *p*> 0.05), but the old male mice with SCI did have more trabecular bone volume than their female counterparts (*t* = 2.95, *p* < 0.01). Significant effects of SCI on bone volume were only evident in the young mice (*t* = 2.94, 2.80, for males and females respectively, *p* < 0.01). There was no effect of SCI on bone volume in the old male and female subjects.

There were also main effects of age (F (1,19) = 144.50, *p* < 0.0001) and sex (F (1,27) = 17.02, *p* < 0.001) on trabecular number (Figure 4C), and significant interactions between sex and age (F (1,19) = 14.20, *p* < 0.005) as well as injury and age (F (1,19) = 5.12, *p* < 0.05). Again, irrespective of sex and injury, old mice had lower trabecular bone numbers than young mice (*t* > 3.91, *p* < 0.0009 for all comparisons). The young females also had lower numbers of trabeculae than the young males (*t* = 3.33, 5.03, for Sham and SCI subjects respectively, *p* < 0.002). There was no difference between trabecular number for old sham males compared with old sham females (*t* = 0.14, *p* > 0.05), but the old SCI male mice did have greater trabecular numbers than old female SCI mice (*t* = 2.11, *p* < 0.01). Significant effects of SCI on trabecular number were only evident in the young female mice (*t* = 2.10, *p* < 0.05). There was no effect of SCI on trabecular number in the young male, old male or old female subjects.

There was a main effect of sex only on trabecular thickness (F (1,17) = 19.03, *p* < 0.001, Figure 4D), with significant interactions between sex and age (F (1,17) = 15.61, *p* < 0.001) as well as sex, injury, and age (F (1,17) = 9.77, *p* < 0.01). The sex effect was driven by the old subjects. Old female mice, with and without SCI, had greater trabecular thickness than the old male mice (*t* = 5.49, 2.40, for Sham and SCI subjects respectively, *p* < 0.05).

Consistent with the other trabecular parameters, for trabecular spacing, there were main effects of age (F (1,19) = 136.90, *p* < 0.0001) and sex (F (1,27) = 27.38, *p* < 0.0001), but no effects of injury or significant interactions (Figure 4E). All young mice had less trabecular spacing than their sex and injury-matched conspecifics (*t* > 5.41, *p* < 0.0001 for all comparisons). Young sham males also had less trabecular spacing than the young sham females (*t* = 2.56, *p* < 0.05), and both the young and old male SCI mice had less spacing than the young and old female SCI mice, respectively (*t* = 4.65, 2.72, *p* < 0.01 for both analyses).

There were also main effects of age (F (1,19) = 126.90, *p* < 0.0001) and sex (F (1,27) = 5.44, *p* < 0.05), and no effects of injury on connective density (Figure 4F). There was also a significant interaction between injury and age on this parameter (F (1,19) = 6.80, *p* < 0.05). All young mice had greater connective density than their sex and injury matched conspecifics (*t* > 4.12, *p* < 0.0005 for all comparisons). Sex effects were seen in the SCI subjects only (*t* = 3.32, 2.27, for young and old SCI subjects respectively, *p* < 0.05). Specifically, an effect of injury was seen in the young females (*t* = 2.16, *p* < 0.05), with SCI decreasing connective density. No effects of sex, age, or SCI were seen on volumetric bone mineral density (Figure 4G).

**Figure 5:**
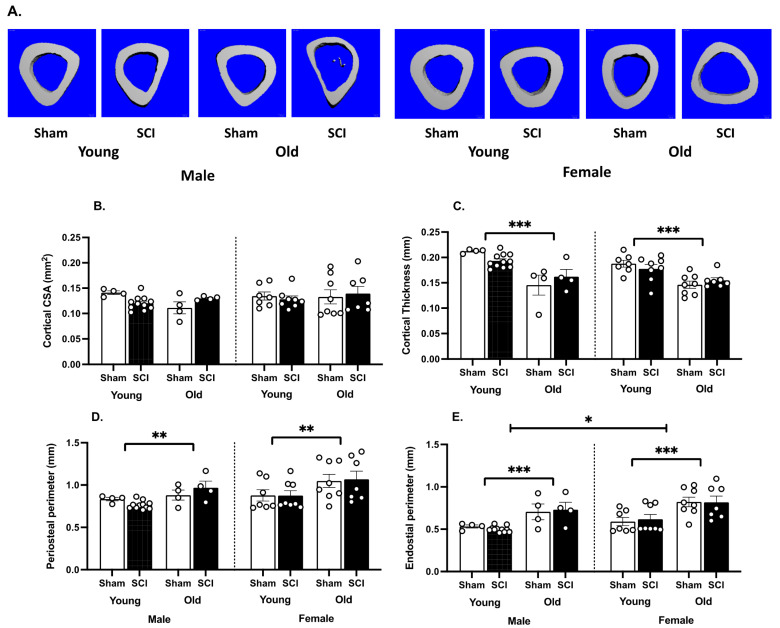
SCI did not affect cortical bone structure after only one month. (**A**). Coronal views of microCT 3D reconstructions of the 50 contiguous slices of the tibial mid-diaphysis from a representative mouse in each group. (**B**). There was also no effect of age or sex on cortical bone cross sectional area. (**C**). Age did, however, decrease cortical thickness and (**D**). increased the periosteal perimeter. (**E**). Both age and sex also affected the endosteal perimeter. The endosteal perimeter increased with age, and was larger in females than males. Means ± SEMs are plotted for each group. Individual subjects are shown with the round, white symbols. n = 4 sham and 5 SCI old males; 4 sham and 11 SCI young males; 8 sham and 7 SCI old females; 7 sham and 8 SCI young females. *** *p* < 0.001, ** *p* < 0.01, * *p* < 0.05.

For cortical bone measurements (Figure 5), only age affected cortical thickness and periosteal perimeter measures (F (1,45) = 45.40, *p*< 0.001; F (1,18) = 14.77, *p* < 0.005, Figure 5C,D, respectively). Young mice had greater cortical thickness than the old mice matched for sex and injury (*t* > 2.16, *p* < 0.05 for all comparisons), and smaller periosteal perimeters for all groups except the sham males (*t* > 2.42, *p* < 0.05 for all comparisons). Both age and sex also affected endosteal perimeters (F (1,18) = 28.91, *p* < 0.0001; F (1,27) = 4.86, *p* < 0.05, respectively, Figure 5E). Post hoc comparisons showed that young mice had greater cortical thickness than the old mice matched for sex and injury for all comparisons, except for the sham males (*t* > 2.91, *p* < 0.01 for all comparisons). There were no effects of SCI on cortical bone at one month post injury.

### 3.3. Open Field Activity and Rearing Were Significantly Correlated with Decreases in Tibial Trabecular Bone Volume

**Figure 6:**
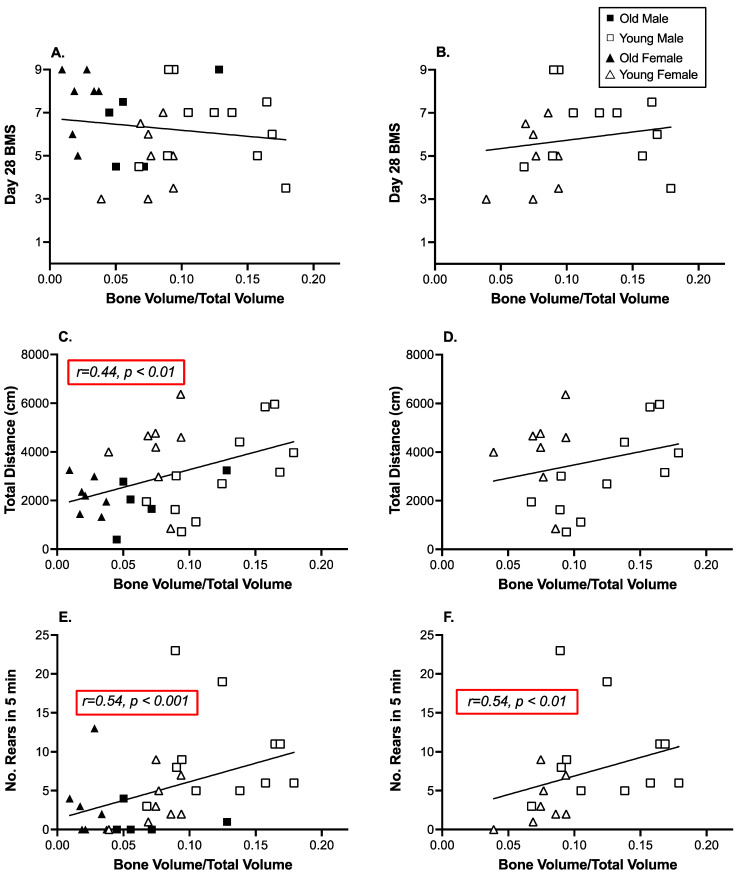
There was no correlation between trabecular bone volume and locomotor function *per se,* but open field activity and rearing were associated with increased bone volume. (**A**). BMS scores collected on Day 28 were not correlated with bone volume across all SCI mice or (**B**). in young SCI mice only. (**C**). There was a strong correlation between open field activity (on Day 21 post injury) and tibia bone volume. (**D**). The correlation between open field activity was not seen in the young mice per se, but was clear for young male mice alone. (**E**). Rearing was also strongly correlated with tibia trabecular bone volume. (**F**). This effect was also clear in the young SCI subjects alone and particularly in the young female subjects. n = 5 SCI old and 11 SCI young males; 7 SCI old and 8 SCI young females.

Pearson’s product-moment correlations were used to examine the relationship between trabecular bone volume and motor activity in the SCI subjects only. With no change in motor function, sham subjects were not included in these analyses. There was no correlation between BMS scores collected on Day 28 and trabecular bone volume (r = −0.14, *p* > 0.05, Figure 6A,B). However, both open field activity and rearing behavior were significantly correlated with trabecular bone volume (r = 0.44, 0.54 respectively, *p* < 0.01, Figure 6C,E). The correlation between open field activity and bone volume was driven by the young male subjects (r = 0.74, *p* < 0.005). Correlations between bone volume and activity were not significant for any other group. The correlation between rearing and bone volume was powered by the young subjects (r = 0.54, *p* < 0.01, Figure 6D,F), although at an individual group level the only correlation that was significant was for the old females (r = 0.74, *p* < 0.05).

## 4. Discussion

Despite the recovery of plantar weight-supported stepping, SCI resulted in decreased tibial trabecular bone volumes in mice one month after injury. The effects of SCI on bone loss were only seen in young mice, irrespective of sex, but this was likely influenced in part by dramatically lower bone volumes seen in older subjects with and without injury producing a floor effect. Trabecular bone volumes were 2–3 fold lower in old mice compared with their young, sex-matched conspecifics. Interestingly, the relationship between trabecular bone volume and activity was also contingent on age. Trabecular bone volume was significantly correlated with open field activity in young males only, and bone volume correlated with rearing in the young, but not old, mice. These data suggest that load and use may reduce bone loss after SCI, but that the effectiveness of physical therapy may be contingent on patient demographics.

The lack of effects of locomotor function on bone volume in the mice, concur with our previous findings in a rat contusion SCI model and with human data. In a previous study of young male rats with a moderate contusion injury we found that recovery of locomotor function did not reduce bone loss after SCI. Despite the recovery of plantar stepping, rats with SCI had lower cancellous bone volume, lower bone formation rate, lower osteoid surface, and higher osteoclast surface at one-month post-injury than age-matched controls [36]. In the current study, there was also no correlation between trabecular bone function and recovered locomotor function, assessed with the BMS scale. Most studies with humans have also shown that standing or walking with assistance is not sufficient to improve bone parameters after SCI (for review see [19]). Instead, the efficacy of activity-based interventions appears contingent on the duration of training and the mechanical strain induced with muscle loading [19]. In the clinical studies, for example, improvements in bone parameters were seen when the number of weekly training sessions were increased, as well as with increased compressive loads during activity (for review see [19]). In the current study, the mice recovered plantar-weight supported stepping, but the SCI might still affect the amount of locomotion that they engage in. For example, both rodents and humans have an increased incidence of depression after SCI [63,64,65,66,67,68,69,70,71], which could reduce motivation to move in the home cage. Rather than locomotor function per se, focusing on activities that place high compressive loads on the bone or increasing the duration of physical activity may be important for re-establishing bone formation after SCI.

Supporting this premise, in the present study we found that increased tibial trabecular bone volume was associated with increased activity levels in an open field and increased rearing. Our interpretation of these data is limited, as activity in the open field is not a complete representation of activity in the home cage and correlations do not denote causation. Nonetheless, these data do suggest that increasing locomotor activity or the load placed on the hindlimbs, below the level of injury, in rearing might protect against bone loss, or increase bone formation, in the mouse SCI model. Notably, the data also suggest that the effects of physical activity may be contingent on the targeted demographic. While tibial trabecular bone volume correlated with increased open field activity and increased rearing in young mice, there was no clear relationship between these variables in the older subjects. Research on the effects of age and physical therapy in older people with SCI is extremely limited, with most studies focused on young adults. There are only two published case reports on older adults with chronic SCI, that were trained three times a week with functional electrical stimulation leg cycle ergometry. For the older adults, training increased lean mass, decreased % body fat, and increased scores on quality-of-life questionnaires. However, it did not improve bone mineral density [72,73]. A recent prospective study of factors associated with a change in bone density in chronic SCI [74], also included participants with a mean age of 55.1 ± 14.4 (SD) years (ranging from 24.7–87.1 years). Interestingly, this large study with 152 participants, found that wheelchair users lost more bone mineral density at the knee than walkers per year, an effect that was independent of age. These data stress the importance of post-injury exercise and potential benefits irrespective of age. However, it is likely that the intensity of the activity and the duration would need to be significantly higher to yield benefits in older, compared to young, adults with SCI [75]. Indeed, even in the able-bodied population, traditional exercise programs seem to be less osteogenic in mature compared to young adults [76]. There appears to be a decline in the sensitivity of bone to mechanical loading with aging. In fact, aging is also associated with declines in the number and function of bone forming osteoblasts, and the differentiation of these cells from multipotent mesenchymal stem cells [77,78,79,80,81,82].

Sex also affects bone loss in the general population, with osteoporosis significantly increased in post-menopausal women. While changes in hormone levels link sex to age in most human studies of bone loss, rats and mice do not experience the abrupt loss of estrogen at menopause, nor do androgen levels seem to decrease with age in male mice [83,84,85]. SCI, however, does produce transient changes in testosterone and estrogen in rodent models, which could differentially affect bone loss [55,86,87,88,89,90]. In the current study, we observed significant effects of sex on trabecular bone volume and number, as well as spacing, thickness, and connective density, irrespective of injury. Young females had lower bone volumes than males, likely corresponding to their lower body weight per se. Indeed, young adult men have almost 25% greater whole body bone mineral content compared with women [91], a difference that can be largely predicted simply based on average height differences between the sexes [92]. Young men also develop greater tibia trabecular bone volume in late puberty, primarily with greater trabecular thickness and trabecular number [93,94,95]. Intriguingly, in humans, these sex differences are only seen in the periphery, and not at central sites [92,96,97]. Our findings in the mouse model concur with the sex differences seen in humans. In the older subjects, however, sex effects were only observed after SCI. After SCI, the older males had increased trabecular bone volume and numbers remaining, increased thickness, and decreased spacing between trabecules compared with the old females, likely due to the fact that so little trabecular bone remained in the aged females to be impacted by SCI (Figure 4A). Interestingly, changes in the trabecular bone with SCI were not related to activity in the older subjects. As can be seen in Figure 3 and Figure 4, rearing and open field activity did not differ between males and females with or without SCI at the older age.

Overall, the data collected in the mouse model of SCI suggest that while simple locomotion is not the key to improving bone integrity after SCI, activities that place high compressive load on the hindlimbs and increased duration of training might improve skeletal integrity. While our data suggest that the benefits of training may be restricted to young adult subjects, there is data to indicate that older adults also benefit from physical rehabilitation in terms of general and potentially bone health in the clinical setting [74]. One limitation of the current study, however, is that our assessments were limited to the acute and subacute stages of SCI. The proposed requisites of training would present challenges in early stages of SCI, as normal weight bearing is often not possible. However, other ways of loading the bone, including electrical stimulation of the muscles, have been explored. In rat models of SCI (complete thoracic transections), electrical stimulation has been shown to reduce bone resorption rates, increase multiple measures of trabecular bone mass, restore cortical mechanical strength, and alter gene expression in bone marrow progenitor cells [98,99]. Specifically, Qin and colleagues found that electrical stimulation increased gene expression for signaling pathways responsible for osteoblast differentiation and function, as well as for the regulation of osteoclasts by cells of the osteoblast lineage. While caution would be required, these data suggest that interventions in the acute phase of injury might reduce the dramatic loss of bone inherent to this stage of SCI. Notably early electrical stimulation could also be applied irrespective of injury severity.

Conceptually, exoskeleton training could also be used in combination with electrical stimulation to increase physical activity and place gravitational load on the lower limbs. A number of bionic exoskeletons are now FDA approved for assisting walking after SCI, including Ekso^TM^, Rewalk^TM^, and Indego^®^ systems, as well as the implanted neuroprostheses, Parastep^®^ 1, which uses functional neuromuscular stimulation (FNS) to generate a majority of the muscular torque required to move and stabilize the lower extremities. These exoskeletal ambulation devices can significantly improve cardiovascular and psychological health [100,101,102,103,104]. However, unfortunately, there is no evidence to suggest that use of these devices can protect against or reverse SCI-induced bone loss. As part of a multicenter study evaluating the effectiveness of the Parastep^®^ 1, Needham-Shropshire et al. [105] assessed bone mineral density in the proximal femur. Sixteen people, at least six months post SCI, completed 32 sessions (three sessions per week over 11 weeks) of Parastep^®^ 1 ambulation training and an additional eight weeks of FNS training There were no changes in bone mineral density in the proximal femur. Similarly, Thoumie et al. [106] reported no beneficial effects of training, with the RGO-II hybrid orthosis, on bone. The lack of effects seen with exoskeleton training could stem from limitations in the duration of walking, which is significantly constrained by fatigue. Indeed, more recently, Karelis et al. [107] reported a tendency for an increase (14.5%) in the bone mineral density of the tibia with use of the battery-powered, motor driven Ekso robotic exoskeleton system, at least three times per week for up to 60 min of free overground walking. Powered exoskeletons that enable increased time spent walking may impact bone. Delayed onset of rehabilitative training might also limit the effectiveness of exoskeleton training. In the Parastep evaluation, training was not initiated until at least six months post injury. Based on the findings of the present study in mice, however, the lower gravitational loads placed on the leg bones when using orthotic devices may be the most critical limitation for osteogenic efficacy. The orthotic components of ambulatory systems are designed to protect the insensate joints and osteoporotic bones of users from possible damage with loads applied during walking [108]. Moreover, ground-reaction forces increase as walking speed increases [109]. Slower walking in orthotic devices would place less compressive force on the bone, and potentially limit the effects of training on osteogenesis. According to Frost’s mechanostat theory [110], there is an optimal range of mechanical loading essential for osteogenesis. The range of mechanical loading that is required to reduce or reverse bone loss after SCI needs to be determined [19].

Currently, there are no clinical guidelines for the prevention or reversal of SCI-induced bone loss. Moreover, interventions that are effective for other forms of osteoporosis have limited efficacy after SCI. While this is not surprising, given the multifaceted etiology of bone loss after SCI, the dramatic bone loss seen after SCI significantly affects psychological and physical well-being. Further exploration of the critical factors contributing to acute bone loss and the development of strategies to improve long-term bone health is an urgent and unmet need for people living with spinal cord injury.

## 5. Conclusions

In sum, commensurate with both preclinical and clinical data, spinal cord injury led to a significant loss of trabecular bone volume below the level of injury. The effects of SCI on bone were only significant in young mice, with the dramatically lower bone volumes seen in older subjects likely masking any effects of injury. Intriguingly, tibial trabecular bone volume in the young SCI mice was significantly correlated with rearing activity. Increased rearing, which would place a high compressive load on the hindlimbs, positively correlated with increased bone volume. These data suggest that while the effectiveness of physical therapy may be contingent on patient demographics, interventions initiated early after SCI that place high compressive load on the hindlimbs may reduce bone loss.

## Data Availability

The data presented in this study are available on request from the corresponding author.
